# Analysis of real‐world capillary blood glucose data to help reduce HbA_1c_ and hypoglycaemia in type 1 diabetes: Evidence in favour of using the percentage of readings in target and coefficient of variation

**DOI:** 10.1111/dme.14972

**Published:** 2022-10-20

**Authors:** Mohammad R. Eissa, Mohammed Benaissa, Tim Good, Zheng Hui, Carla Gianfrancesco, Carolin Ferguson, Jackie Elliott

**Affiliations:** ^1^ Department of Electronic and Electrical Engineering University of Sheffield Sheffield UK; ^2^ Department of Diabetes and Endocrinology Sheffield Teaching Hospitals NHS FT Sheffield UK; ^3^ Department of Oncology and Metabolism University of Sheffield Sheffield UK

**Keywords:** coefficient of variation, glucose retest, hyperglycaemia, hypoglycaemia, proportions in target range, time in range

## Abstract

**Aims:**

To examine real‐world capillary blood glucose (CBG) data according to HbA_1c_ to define proportions of CBG readings at different HbA_1c_ levels, and evaluate patterns in CBG measurements to suggest areas to focus on with regard to self‐management.

**Methods:**

A retrospective analysis stratified 682 adults with type 1 diabetes split into quartiles based on their HbA_1c_. The proportions of results in different CBG ranges and associations with HbA_1c_ were evaluated. Patterns in readings following episodes of hyperglycaemia and hypoglycaemia were examined, using glucose to next glucose reading table (G2G).

**Results:**

CBG readings in the target range (3.9‐10 mmol/L) increase by ~10% across each CBG quartile (31% in the highest versus 63% in the lowest quartile, *p* < 0.05). The novel G2G table helps the treatment‐based interpretation of data. Hypoglycaemia is often preceded by hyperglycaemia, and vice‐versa, and is twice as likely in the highest HbA_1c_ quartile. Re‐testing within 30 min of hypoglycaemia is associated with less hypoglycaemia, 1.6% versus 7.2%, *p* < 0.001, and also reduces subsequent hyperglycaemia and further hypoglycaemia in the proceeding 24 h. The coefficient of variation, but not standard deviation, is highly associated with hypoglycaemia, r = 0.71, and a CV ≤ 36% equates to 3.3% of CBG readings in the hypoglycaemic range.

**Conclusions:**

HbA_1c_ <58 mmol/mol (7.5%) is achievable even when only ~60% of CBG readings are between 3.9–10 mmol/L. Examining readings subsequent to out‐of‐range readings suggests useful behaviours which people with type 1 diabetes could be supported to adhere to, both in a clinic and structured education programmes, thereby decreasing the risk of hypoglycaemia whilst also reducing hyperglycaemia and improving HbA_1c_.

**Novelty Statement**

**What is already known?**

- Guidelines suggest pre‐meal and pre‐bed capillary blood glucose (CBG) targets. Continuous glucose monitoring (CGM) targets are based on time in range and coefficient of variation (CV).

**What this study has found?**

- New targets based on the proportion of in‐range, and CV of CBG data.
- Re‐testing after hypoglycaemia results in less hypoglycaemia and less hyperglycaemia.
- A novel diagnostic tabulation, Glucose to Glucose (G2G), facilitates identifying useful behaviours to amend.

**What are the implications of the study?**

- Behaviours and targets to address in clinics, or structured education programmes, have been identified to help people with type 1 diabetes reduce hypoglycaemia, whilst also improving glycaemic control, irrespective of the method of glucose monitoring.

## 1 INTRODUCTION

It is increasingly recognized that the use of HbA_1c_ alone is insufficient for describing the status of diabetes management.^1^, ^2^, ^3^ More importantly, whilst there is a positive association between the understanding of HbA_1c_ levels and self‐care behaviours, only ∼25% of people with diabetes have good knowledge of the HbA_1c_ metric.^4^, ^5^, ^6^ Therefore, there is a need for more meaningful parameters that are easy to understand. With the advent of increasing access to continuous glucose monitoring (CGM), there has been a shift towards using time in range, advocating the interstitial glucose target of 70% of the time between 3.9 and 10 mmol/L, with <4% of the time under 3.9 mmol/L.^7^ This approach has the advantage of aiding the evaluation of changes in self‐management in a shorter time frame, for example, a weekly basis,^8^, ^9^ as opposed to the inherent 3 monthly nature of HbA_1c_. Whilst CGM is becoming more commonplace, it is by no means universal, and for those living in developing countries, it may be quite some time before it is widely available. Therefore, for those using capillary blood glucose measurements (CBG), a similar approach that provides more informative feedback than HbA_1c_ alone would be advantageous. Hence, analysing routinely collected CBG data could aid in setting more tangible targets for those utilizing this method of glucose monitoring.

The percentage of readings in the target range has been explored using data from the Diabetes Control and Complications Trial (DCCT) and shows that the risk of progression to retinopathy is increased by 64% for each 10% decrease in time in target.^10^ However, this analysis consisted of only 7‐point profiles on 1 day of a 3‐month period as opposed to consecutive daily CBG results.^10^ Avari et al.^11^ compared CBG and CGM data, but the population studied in the paper was not typical of real‐world clinics as the median HbA_1c_ was 53 mmol/mol (7%). Sivasubramaniyam et al.^12^ analysed 14–28 days of data from 201 people using insulin pumps (CSII). The authors suggested a shift of focus from the glycaemic target range to proportions in the target. Nevertheless, the thresholds of these proportions whereby various achievable HbA_1c_ levels are obtained remain under‐studied for the majority of people who use multiple daily injections (MDI).

On a reading‐by‐reading basis, the achieved proportions of various CBG ranges are driven by actions taken either to correct/treat an out‐of‐range CBG or to maintain an in‐range CBG. Therefore, a representation of CBG levels providing insight into potential behaviours that are leading to diabetes self‐management shortfalls, such as under treatment of hypoglycaemia or over‐correction of hyperglycaemia would be helpful. Another potential metric is the fluctuation in CBG, which is yet to be established as an independent risk factor for diabetes complications.^13^, ^14^

This study examines these understudied areas by investigating the relationship between CBG and HbA_1c_, through the retrospective analysis of routine clinical data of people with type 1 diabetes using MDI and CSII. For those undertaking CBG, it provides a recommendation as to the proportion of readings in the target range to aim for, which can be reviewed on a weekly, or monthly basis. More rapid feedback could increase engagement and motivation and may be perceived as a more tangible target than HbA_1c_, thereby helping to reduce the associated anxiety and stress of diabetes self‐management.^15^, ^16^ Additionally, CBG readings following an out‐of‐range reading are explored in a new format called Glucose to next Glucose (G2G) tables. This tool can help to illustrate the cycle of over‐treatment of hypoglycaemia leading to hyperglycaemia, which in turn may lead to over‐correction and another hypoglycaemic episode, a roller‐coaster type effect. This novel representation of data may aid the promotion of useful self‐management behaviours.

## 2 MATERIALS AND METHODS

A retrospective, cross‐sectional analysis was performed to investigate the glycaemic patterns and behaviours of people in the real world, residing in a large UK city. People were eligible for inclusion if CBG readings were available in the 12 weeks prior to their latest HbA_1c_ lab test, between the 4th quarter of 2013 to the 4th quarter of 2015, and if BMI, age, sex, ethnicity, and age at diagnosis were known, leading to complete case analysis of the data. This cohort attended out‐patient clinics at Sheffield Teaching Hospitals NHS Foundation Trust and was routinely advised to test before meals, before bedtime, and at times of suspected hypoglycaemia. CBG meters were downloaded, and HbA_1c_ lab tests were performed every 3–12 months. For each participant, CBG readings were extracted for 12 consecutive weeks prior to their latest HbA_1c_, and demographic data were collected from their hospital records. People were also advised to retest CBG at 15 min intervals following a hypoglycaemia event until the CBG was above 4 mmol/L,^17^ but any reading taken within the 30 min following a hypoglycaemia event was excluded from the analysis to avoid oversampling bias. Ethical approval under STH Ref 20,586 was granted for the use of the data.

### 2.1 Statistical analysis

Participants' results were stratified based on the HbA_1c_ interquartile ranges: <58 mmol/mol (<7.5%) (Q1), 58–65 mmol/mol (7.5%–8.1%) (Q2), 66–74 mmol/mol (8.2%–8.9%) (Q3) and >74 mmol/mol (>8.9%) (Q4). Unless stated otherwise, summaries are provided on a patient‐level basis, using means and standard deviations. Fluctuations in CBG were examined by both standard deviation SD (a measure of absolute variation) and coefficient of variation CV (a measure of relative variation). CBG ranges were stratified as shown in Table 1.

**TABLE 1:** CBG categories, hypoglycaemia results were stratified based on the International Hypoglycaemia Study Group guidelines^24^

CBG Category	Range (mmol/L)
Hypoglycaemia	
Clinically significant hypoglycaemia	<3.0
Alert value hypoglycaemia	3.0–3.8
Euglycemia	
In the target‐range	3.9–10.0
Hyperglycaemia	
Mild hyperglycaemia	10.1–13.9
Significant hyperglycaemia	>13.9

For each of the HbA_1c_ quartiles, the following summaries were produced: proportion of data in the five CBG ranges; associations between significant and mild hyper‐ and hypo‐glycaemia on HbA_1c_; frequency of CBG measurements and its association with HbA_1c_; differences in glucose levels by HbA_1c_ quartile; frequency of over‐correction of hyperglycaemia; frequency of over‐treatment of hypoglycaemia; and frequency of re‐testing CBG after hypoglycaemia. Additionally, the relationship between pairs of CBG readings is displayed in novel G2G tables.

The data were not normally distributed (visual examination of the data) and therefore groups were compared where applicable using either the nonparametric Mann–Whitney or Kruskal‐Wallis tests. Robust linear regression^18^ was utilized for the regression analysis to account for outliers. Hierarchical linear regression was conducted to examine the variance explained by covariates, outcome was HbA_1c_ and covariates were frequency of CBG, SD of CBG and mean of CBG. Logistic regression results are reported where applicable (Outcome was if HbA_1c_ < 58 mmol/mol (7.5%) is achieved for frequency ranges of CBG testing). The statistical analyses were carried out using Python ‘statsmodels’ package (v0.9.0) and R (v3.3.3).

## 3 RESULTS

Approximately 1500 people with type 1 diabetes attended outpatient clinics at Sheffield Teaching Hospitals NHS Foundation Trust attended between the 4th quarter of 2013 and the 4th quarter of 2015, of which 843 had their CBG readings uploaded onto the hospital system. The subset of 682 people had CBG readings within 12 weeks prior to their latest HbA_1c_ lab test and available data for the key patient characteristics, and formed the analysis population for this study, contributing a total of 211,929 CBG readings. 83.9% of the participants were on MDI and 16.1% on CSII, characteristics are shown in Table 2.

**TABLE 2:** Characteristics of the participants, HbA_1c_ results and accompanying CBG results of the 90 days prior to the HbA_1c_ test

*N*	682
Age (years)	46.5 ± 17.6
BMI (kg/m^2^)	26.5 ± 5.07
Duration of type 1 diabetes (years)	23.6 ± 15.2
Ethnicity (%)	90.2% white Caucasian, 9.8% others
Sex (female)	51.2%
HbA_1c_ (mmol/mol and (%))	66.4 ± 13.2 (8.2% ± 1.2)
CBG levels (mmol/L)	10.5 ± 2.4
Number of days with CBG	84.5 ± 2.4 (median 86 [83–86])
Frequency of CBG (times/day)	3.7 ± 1.3 (median 3.4 [2.5–4.4])
Standard deviation of CBG (mmol/L)	4.7 ± 1.3
Coefficient of variation of CBG (%)	44.9 ± 8.4

*Note*: Data are mean ± SD or median (25–75 percentiles). Abbreviation: CBG, Capillary Blood Glucose.

### 3.1 Percentage of readings in the target‐range

**FIGURE 1:**
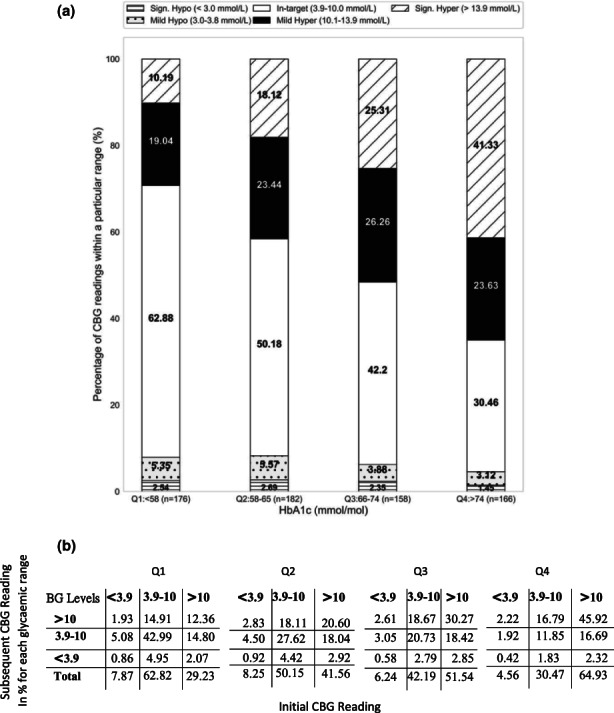
Proportions of 211,929 readings in various ranges of CBG: (a) Percentages of readings across HbA_1c_ quartiles and *n* is the number of participants; in the target range 3.9–10 mmol/L (white), significant hyperglycaemia >13.9 mmol/L (diagonal stripes), mild hyperglycaemia 10.1–13.9 mmol/L(black), alert value hypoglycaemia 3–3.8 mmol/L (dotted) and significant hypoglycaemia <3 mmol/L(horizontal stripes) and (b) Glucose to glucose (G2G) tables representing the changes between an initial CBG reading in the columns and the next CBG reading in the rows indicated by the proportion of readings (%), split into hyperglycaemia >10 mmol/L, hypoglycaemia <3.9 mmol/L and in the target range 3.9–10 mmol/L.

Figure 1a shows the percentage of readings in the various glucose ranges differ significantly across the HbA_1c_ quartiles, (*p* < 0.05). The readings in the target range of 3.9–10 mmol/L increase by about 10% for each lower quartile of HbA_1c_ readings, whilst significant hyperglycaemia >13.9 mmol/L decreases by approximately 10% across each quartile. The percentage increase in hypoglycaemic readings from highest to lowest HbA_1c_ quartile was 4.56% (Q4) to 7.87% (Q1) (*p* < 0.05). For the percentage ranges presented in median instead of mean refer to Figure S4.

### 3.2 G2G reading

This analysis examined the change in CBG range from one CBG reading to the next. Before mealtime, if a CBG is in the target range and the insulin doses have been calculated correctly, then the next pre‐meal reading should remain in the target range. If the CBG is outside the target range, then either extra‐quick acting insulin to correct hyperglycaemia, or consumption of hypoglycaemia treatment to raise a low CBG is required. In the G2G tables (Figure 1b), the columns represent the category of the initial glucose reading (<3.9, 3.9–10 or >10 mmoL/L), and the rows represent the subsequent glucose category. As shown in the G2G tables, even those in HbA_1c_ Q1 (<58 mmol/mol [<7.5%]) only have a reading in the target range, followed by the next reading in the target range ~ 43% of the time (See Figure S3 for results when only participants with 3–6 tests/day were considered). This ability to keep reading in the target range decreases by ~10% for each of the subsequent quartiles. (Median of the percentages is presented in Figure S4).

Additionally, those in Q1 are ~2‐fold more likely to accurately treat a hyperglycaemic reading, in 50.7% of occasions (14.8% out of a total of 29.2% of readings), compared to only 25.7% of occasions for those in Q4 (16.7% out of a total of 64.9% of readings). Likewise, Q1 participants are 1.5 times more likely to accurately treat a hypoglycaemic episode so that the next reading is in the target range, 64.5% (5.1% out of a total of 7.9% of readings) versus 42.1% (1.9% out of a total of 4.6% of readings) for those in Q4 (>74mmolmol [>8.9%]).

**FIGURE 2:**
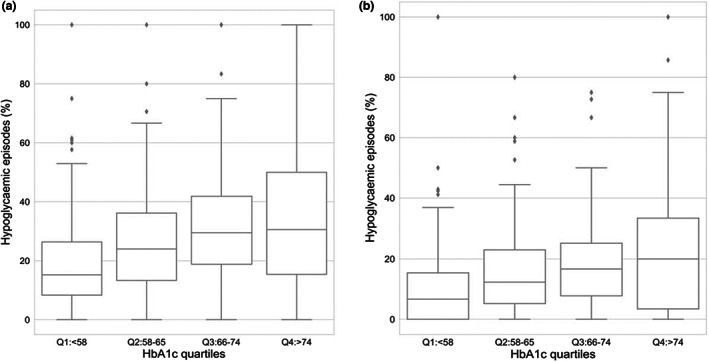
Percentages of hypoglycaemic episodes resulting from over‐correction of hyperglycaemic excursions. The median and interquartile ranges are shown using box and whisker plots. The outliers are shown as individual points (a) over‐correction of hyperglycaemia >10 mmol/L, (b) over‐correction of hyperglycaemia >13.9 mmol/L.

**FIGURE 3:**
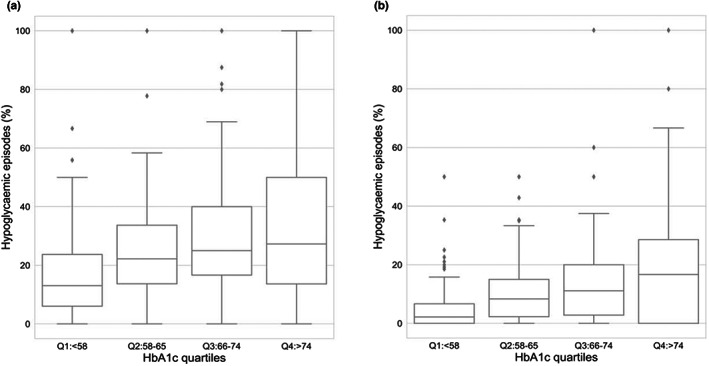
Percentages of hypoglycaemic episodes resulting in a hyperglycaemic excursion by over‐treating. The median and interquartile ranges are shown using box and whisker plots. The outliers are shown as individual points (a) over‐treated hypoglycaemic episode resulting in hyperglycaemia >10 mmol/L, (b) Over‐treated hypoglycaemic episode resulting in hyperglycaemia >13.9 mmol/L.

Furthermore, as shown in Figure 2, we examined the prevalence of hypoglycaemic episodes following a hyperglycaemic reading >10 mmol/L(Figure 2a), perhaps because of over‐correction with quick acting insulin. It increases by ~2‐fold from Q1 (18.6%) to Q4 (35.2%), and likewise when the hyperglycaemic threshold is >13.9 mmol/L (Figure 2b), (9.8% in Q1, and 24.8% in Q4). The reverse of this effect: the prevalence of hypoglycaemic readings resulting in hyperglycaemia perhaps as a result of consuming more carbohydrates than required was determined, i.e. rebound hyperglycaemia. Once again, this undesirable outcome was approximately twice as likely for those in Q4, as opposed to Q1, with 31.8% of hypoglycaemic episodes resulting in hyperglycaemia (>10 mmol/L) in Q4, compared to 16.2% in Q1 (Figure 3a). The pattern was even more marked for the significant hyperglycaemia level of >13.9 mmol/L, where rebound hyperglycaemia was fourfold more likely, 18.9% versus 4.5% respectively for Q4 as opposed to Q1 (Figure 3b).

### 3.3 Fluctuations in CBG readings

**FIGURE 4:**
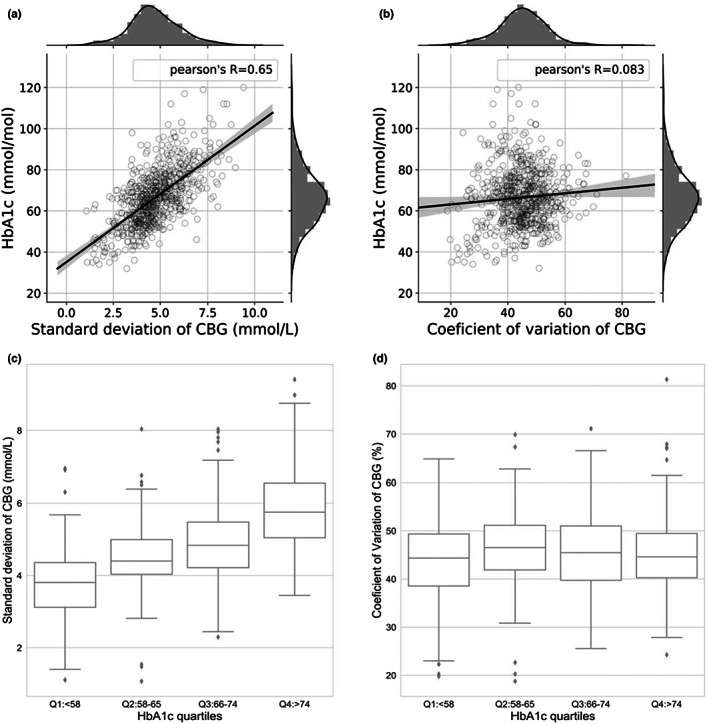
(a, b) Variation of blood glucose levels vs HbA_1c_, using SD and CV respectively. (c, d) Variation in each HbA_1c_ quartile was presented as median and interquartile ranges using box and whisker plots, using SD and CV respectively. The potential outliers are shown as individual points.

Variations in CBG readings were compared between HbA_1c_ quartiles using SD and CV. The CBG fluctuation measured by SD shows a strong positive correlation with HbA_1c_ (0.65, *p* < 0.001, see Figure 4a), increasing as HbA_1c_ quartile increases (Q1 = 3.7, Q2 = 4.5, Q3 = 4.9, Q4 = 5.9, *p* < 0.001, see Figure 4c). Utilizing robust linear regression^18^ analysis showed an increase in 1 mmol/L in CBG SD resulting in an increase in 6.6 mmol/mol in HbA_1c_ (*p* < 0.001). Conversely, the CV of the daily CBG readings showed only a weak correlation with HbA_1c_ (0.083, *p* = 0.03, see Figure 4b) and unlike SD, CV is constant across the quartiles (Q1 = 43.1%, Q2 = 46.6%, Q3 = 45.1%, Q4 = 45.1%, *p* > 0.05, see Figure 4d). Therefore, the relative variability (CV) of CBG readings is not a significant predictor of HbA_1c_, whereas the absolute variability (SD) is (Figure 4).

**FIGURE 5:**
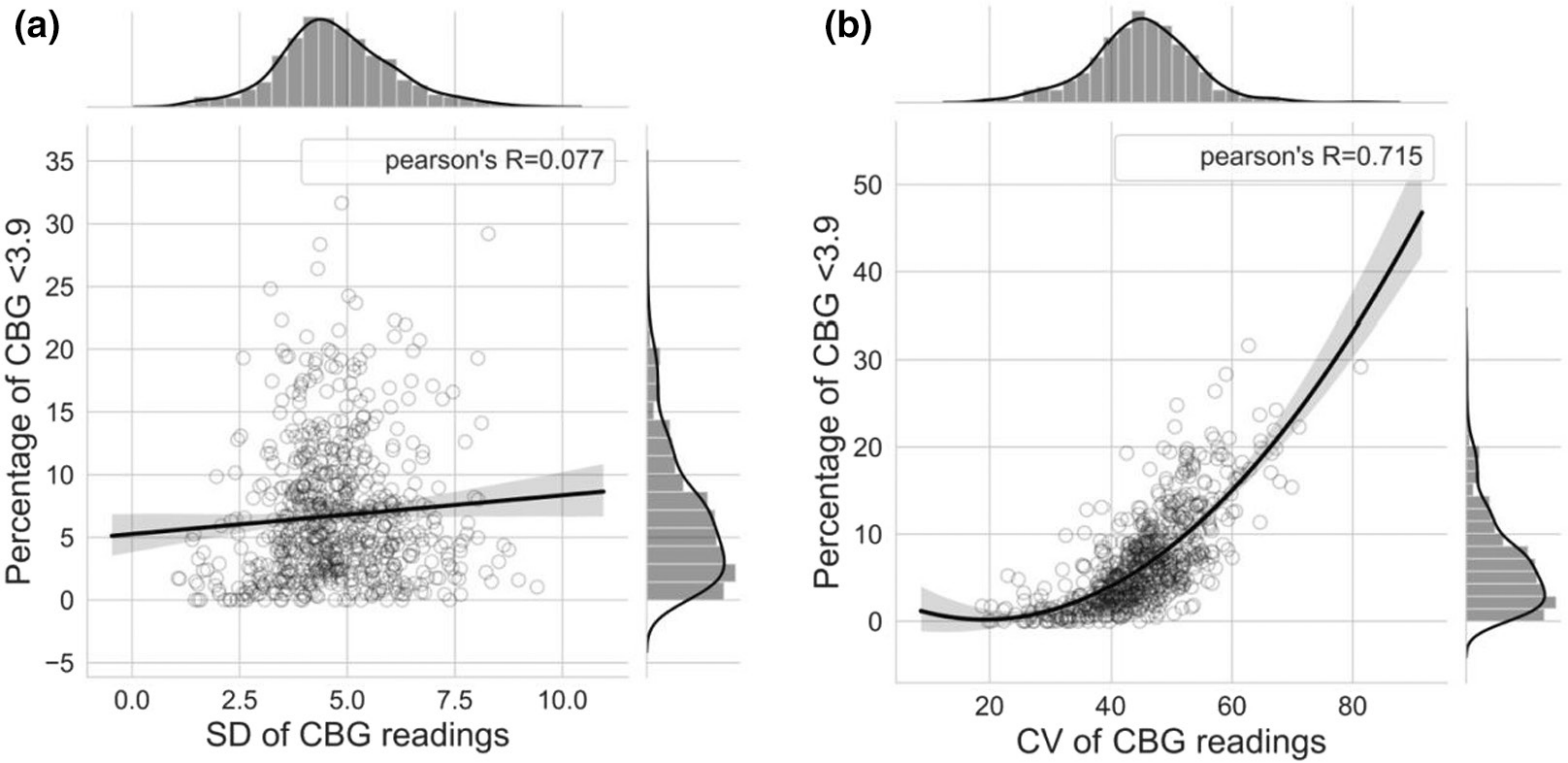
Percentage of hypoglycaemic episodes in relation to fluctuations in daily CBG measurements: (a) Standard deviation (absolute dispersion) of CBGs compared to hypoglycaemia percentage (b) Coefficient of variation (relative dispersion) of CBGs compared to hypoglycaemia percentages, the quadratic equation is $\%\text{Hypoglycaemia}=0.0089\;{\left(\frac{\mathrm{CV}}{100}\right)}^{2}–0.342\frac{\mathrm{CV}}{100}+3.463$.

However, as Figure 5b shows, CV is strongly correlated with the percentage of hypoglycaemic episodes <3.9 mmol/L, r = 0.72. Furthermore, For clinically significant hypoglycaemic episodes <3 mmol/L the association was r = 0.63. The relationship between hypoglycaemia and CV in our dataset is best described by a quadratic non‐linear regression, where $\%\text{Hypoglycaemia}=0.0089\;{\left(\frac{\mathrm{CV}}{100}\right)}^{2}–0.342\frac{\mathrm{CV}}{100}+3.463$ (utilizing ANCOVA analysis of various models). When CV ≤ 36% is selected (as recommended when examining CGM data^7^) the percentage of readings in the hypoglycaemic range (<3.9 mmol/L) equates to 3.3%. Variability in CBG readings as measured by SD is not correlated with hypoglycaemia (r = 0.077, see Figure 5a).

### 3.4 Frequency of CBG readings

**FIGURE 6:**
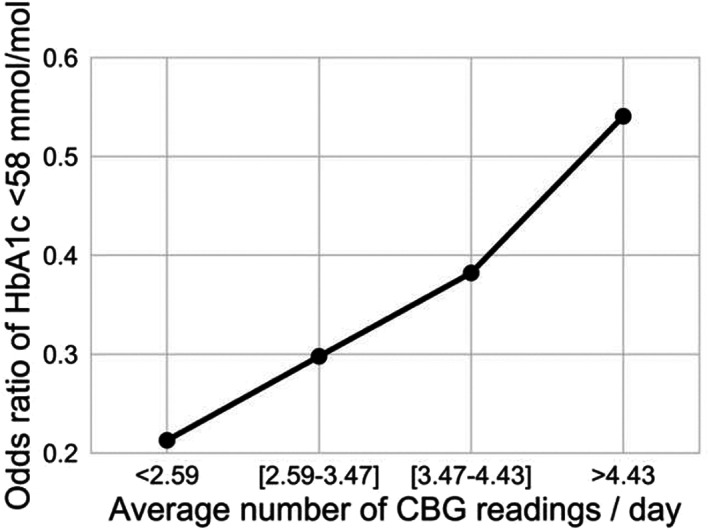
Frequency of CBG testing and relationship to HbA_1c_: Odds ratios of achieving HbA_1c_ <58 mmol/mol (7.5%) per quartile of CBG testing frequency.

As expected, the increased frequency of CBG tests is associated with a decrease in HbA_1c_ levels (Figure S1). Utilizing robust linear regression analysis showed an increase of one test/day reduces HbA_1c_ levels by 2.3 mmol/mol (*p* < 0.001). However, the frequency of CBG measurements can only explain 5% of the variation in HbA_1c_ as an outcome variable (*R* ^2^ = 0.05). With the addition of CBG and SD as controlling factors, the frequency of CBG measurements remained significant (*p* < 0.05). Furthermore, logistic regression of the stratified frequency of CBG showed an increase in odds of achieving an HbA_1c_ of <58 mmol/mol (<7.5%) from 0.22 to 0.54 when performing more than 4.4 tests per day, compared to less than 2.6 test per day (*p* < 0.05) (Figure 6) (cut‐offs based on interquartile ranges of the distribution). The frequency of measurement only has a weak negative correlation with variability r = 0.19 for SD and r = −0.014 for CV. Also, the correlation between CBG frequency and hypoglycaemia was weak with r = 0.073 (*p* = 0.056).

### 3.5 Retesting after Hypoglycaemia

Despite self‐management recommendations to re‐test after treating hypoglycaemia, most hypoglycaemic readings (~75%) were not followed by a re‐test (within 30 min). The hypothesis that a retest affects the chances of a further episode of hypoglycaemia in the following 24 h was examined. Data separation produced three distinctive clusters: ‘mostly’ re‐test (>60% of their hypoglycaemic episodes), ‘sometimes’ re‐test (between 15% and 60%), and ‘rarely’ re‐test (<15%) (Interquartile ranges did not provide a reasonable separation as most of the participants do not retest). Although their HbA_1c_ levels were similar (*p* = 0.93), the ‘mostly’ re‐testing group had the lowest prevalence of hypoglycaemic episodes, and they also exhibited the lowest variation as measured by both SD and CV (*p* < 0.01), Table 3. Irrespective of usual re‐testing behaviour, if a re‐test was performed then the chances of a hypoglycaemic episode in the following 24 h were similar (*p* = 0.38), presumably as further carbohydrate was consumed if necessary. However, the lack of a follow‐up re‐test on any individual occasion resulted in twice as much risk of a hypoglycaemic reading the next day in the ‘rarely’ re‐testers, compared to the ‘mostly’ re‐test group (7.7% vs. 3.6%, *p* < 0.001). The ‘mostly’ retesting group are also less likely to over‐treat their hypoglycaemic episodes to cause hyperglycaemia (>13.9 mmol/L), *p* = 0.04. In other words, the ‘mostly’ re‐testing group, even if they missed the retest, experienced both fewer subsequent hypoglycaemic episodes and less rebound hyperglycaemic episodes (Figure S2).

**TABLE 3:** The CBG and HbA_1c_ outcomes of groups based on retesting ‘rarely’ (<15%), ‘sometimes’ (15%–60%) and ‘mostly’ (>60%) within 30 mins post‐hypoglycaemia (CBG <3.9 mmol/L)

Re‐test habits	Mostly	Sometimes	Rarely	*p*‐value
Re‐test percentage	>60	15–60	<15	
Number of participants	(*n* = 44)	(*n* = 132)	(*n* = 506)	
Average CBG (mmol/L)	10.7 ± 3.4	10.6 ± 2.2	10.4 ± 2.3	0.51
SD	3.8 ± 1.5	4.9 ± 1.2	4.7 ± 1.2	<0.01
CV (%)	35 ± 8	46 ± 9	45 ± 7	<0.001
Number of days with CBG	84.6 ± 2.1	84.6 ± 2.4	84.4 ± 2.5	0.58
Frequency of CBG/day	3.8 ± 1.7	3.9 ± 1.5	3.5 ± 1.2	0.046
HbA_1c_ (mmol/mol)	67.3 ± 19.7	65.8 ± 13.0	66.5 ± 13.2	0.93
% of readings <3.9 mmol/L	1.6 ± 2.3	6.3 ± 5.1	7.2 ± 5.3	<0.001
% of readings <3.9mmo/L in the next 24 h if re‐test performed	2.6 ± 2.8	5.0 ± 5.9	7.3 ± 10.7	0.38
% of readings <3.9 mmol/L in the next 24 h if re‐test not performed	3.6 ± 5.3	6.3 ± 6.0	7.7 ± 6.4	<0.001
% of readings >10 mmol/L if re‐test performed	25.9 ± 18.2	34.1 ± 19.9	31.0 ± 21.9	0.16
% of readings >10 mmol/L if no re‐test performed	33.5 ± 28.1	43.5 ± 20.9	44.1 ± 20.2	0.051
% of readings >13.9 mmol/L if re‐test performed	12 ± 12.6	18.3 ± 17.8	14.4 ± 16.4	0.035
% of readings >13.9 mmol/L if no re‐test performed	14.7 ± 20.5	22.6 ± 18.9	21.4 ± 16.2	0.045

*Note*: Data are mean ± SD, *n* is counts and *p* < 0.05 is significant.

## 4 DISCUSSION

This assessment of a large real‐world dataset illustrates the potential utility of the percentage of readings in the range to be utilized for those people undertaking CBG measurements. It shows that like studies of CGM data not all readings need to be in the target range. For each improved HbA_1c_ quartile the percentage of CBG readings in‐target increases from 31% in those with the highest HbA_1c_s, to 63% in those with the lowest HbA_1c_. HbA_1c_ alone is insufficient to describe the status of glucose control as it provides no measure of the proportion of hypoglycaemia or glycaemic variability, and often is unavailable at the time of the clinical appointment. Therefore, the proposed mid‐term targets using a percentage of readings in the target range is a possible way to simplify the process and provide objective feedback. Encouraging people with type 1 diabetes to aim for at least 60%–70% of their pre‐meal and pre‐bed readings to be in the target range is a realistic goal. Importantly, it potentially reduces anxiety and feelings of failure when a hyperglycaemic reading is encountered.^15^, ^16^ It also allows continuous tracking of progress, but on a more frequent and less cumbersome basis than HbA_1c_. This approach is one component currently being tested as part of the DAFNE*plus* randomized control trial.^19^

Previous work by Sivasubramaniyam et al.^12^ suggested a shift of focus from the target range to the proportion in the target. However, their study has several limitations. Their conclusion was based on CBG data for 14–28 days in CSII users only, accounting for only a fraction of the 12‐week HbA_1c_ period. The study was performed on a small sample, especially in the group with HbA_1c_ > 74 mmol/mol (8.9%) (*n* = 36). Furthermore, the study does not indicate any adjusted proportions of hypoglycaemic readings due to a re‐test; hence, perhaps resulting in an overestimation of the percentage of separate hypoglycaemic episodes, and an underestimation of the hyperglycaemia and in‐target range percentages. In comparison, our study is larger and more comprehensive examining 12 weeks of data preceding the HbA_1c_ test, predominantly in those using MDI. Our complete case analysis of the data may have introduced a bias in terms of the sample representation, as not all people with type 1 diabetes brought blood glucose meters to the clinic. However, the included population size and real‐world routinely collected data covered a broad range of HbA_1c_s. Our data, in common with previous studies,^20^, ^21^, ^22^ shows that increasing frequency of CBG tests is associated with a significant, but small, HbA_1c_ improvement.

The novel G2G tables show how the initial CBG is related to the next CBG for the populations within the quartiles, but equally could be generated for an individual. Namely, attaining a glucose reading in the target range and keeping it there is challenging, and even those in the lowest HbA_1c_ quartile only achieve this on average 43% of the time. It also highlights how often the over‐treatment of hypoglycaemia and over‐correction of hyperglycaemia may create adverse effects, analogous to a roller‐coaster type effect in glucose levels, which patients would prefer to avoid.^23^ In the quartiles with a HbA_1c_ level > 58 mmol/mol (7.5%), approximately 30% of their hypoglycaemic episodes are preceded by hyperglycaemic readings and so could be the result of over‐correction; and about 30% of their hypoglycaemic episodes are followed by hyperglycaemic readings, showing possible over‐treatment with carbohydrate or under‐dosing of insulin with the next meal. Although these groups experience fewer hypoglycaemic episodes overall, the likelihood of subsequent hyperglycaemia and therefore maintenance of raised HbA_1c_ is higher. Those people in the lowest HbA_1c_ quartile seem to be better skilled in the management of such situations, and thus this may be a distinguishing factor of their relatively better diabetes management. Hence, transferring such skills to higher risk groups may help them manage these situations more objectively. This also demonstrates where potential technological advances in terms of automated decision support tools may play a role, for those using CBG or CGM. The G2G analysis could be implemented as a tool in glucose meters and diabetes management apps/software to identify some actionable key messages.

Importantly, we have demonstrated that lower variation in CBG as measured by CV is associated with less hypoglycaemia and that a cut‐off of 36% is a useful marker of low variability in both CBG as well as CGM^7^ data, as this level was also associated with <4% of CBG readings being under 3.9.

Analysing the re‐testing behaviour of the participants in the event of hypoglycaemia showed that although the HbA_1c_ levels of the re‐test groups were similar, the mostly re‐testing group experience approximately half the number of hypoglycaemic episodes. Re‐testing has a positive effect in the following 24 h to reduce the chance of a further hypoglycaemic episode, irrespective of the group. However, those with the highest retesting habit, even when they missed a re‐test, experienced fewer hypoglycaemic episodes in the following 24 h, and less rebound hyperglycaemic excursions too. The reason for this is unknown but could possibly be because they habitually took the correct amount of carbohydrates for an episode of hypoglycaemia. These findings indicate that perhaps a greater focus is needed to help people with diabetes understand the importance and benefits of retesting CBG post‐treatment of hypoglycaemia, and again would apply equally to those on CGM.

## 5 CONCLUSION

This retrospective analysis allowed correlations of CBG patterns with clinical outcomes and as a result, provides new insights into useful behaviours and less useful practices to be addressed at clinic appointments and by structured education programmes; for example, over‐treating hypoglycaemic episodes and over‐correcting higher glucose levels. The G2G representation shows the quality of decision making and could be used by individuals, clinicians, or metrics for trials. To our knowledge, this is the first study to provide evidence for measuring CV in CBG data to assess hypoglycaemia risk and the usefulness of retesting after a hypoglycaemic episode to reduce subsequent out‐of‐range glucose levels. It also provides evidence to support the recommendation of short‐ and mid‐term individualized goals based on the proportion of CBG tests in the target range to ultimately improve glycaemic control.

## CONFLICT OF INTEREST

J.E. has served on advisory boards and/or received speaker fees for Abbott, DEXCOM, Eli Lily, Insulet, NovoNordisk, and Sanofi S.A., however, has no potential conflicts of interest relevant to this article. All other authors have no conflicts of interest to declare.

## Supporting information


Figure S1
Figure S2
Figure S3
Figure S4
