# Supplementary material for: Analysis of real‐world capillary blood glucose data to help reduce HbA1c and hypoglycaemia in type 1 diabetes: Evidence in favour of using the percentage of readings in target and coefficient of variation
Source: Diabet Med. 2022 Oct 20;40(2):e14972. doi: 10.1111/dme.14972 (PMC10091810; doi:10.1111/dme.14972)
Supplement: Supplementary file 1 — Figure S1 Figure S2 Figure S3 Figure S4 [file DME-40-0-s001.pdf]

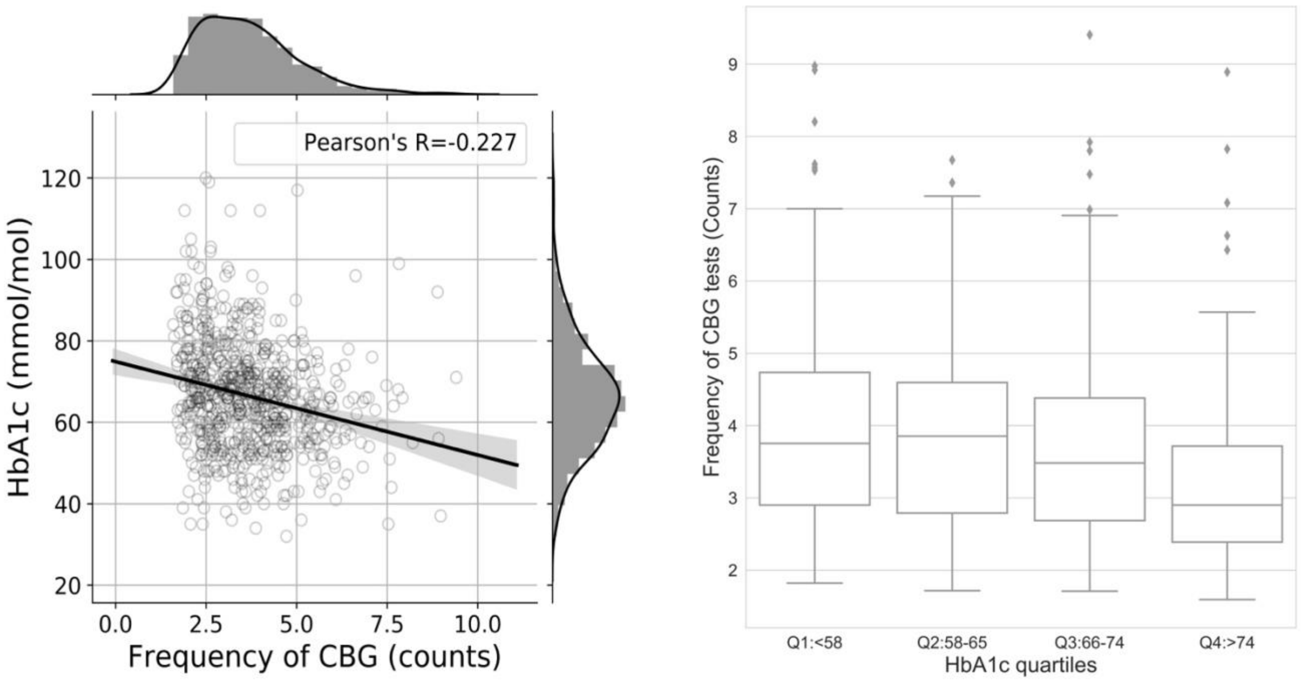

(A) (B)

Figure S1: The relationship of frequency of blood glucose measurements with HbA1c (A) Linear relationship of frequency of CBG measurements with HbA1c (B) Frequency of CBG measurements in each HbA1c quartile presented as median and interquartile ranges using boxplot. The outliers are shown as individual points.

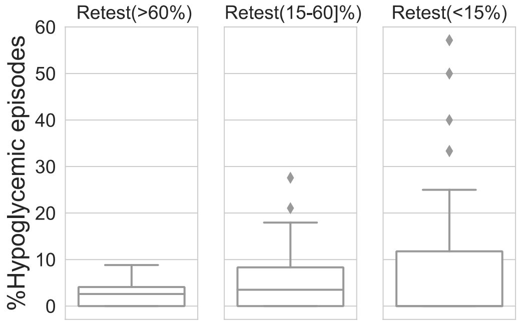

(A)

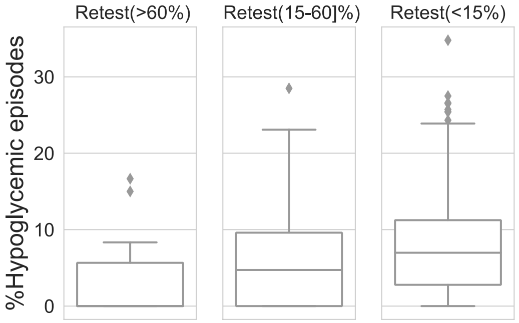

(B)

Figure S2: The hypoglycemia treatment behavior **(A)** the percentages of hypoglycemic episodes occurring in the following 24 hours of a hypoglycemia reading in the case where the treated hypoglycemic reading was followed by a retest **(B)** the percentages of hypoglycemic episodes occurring the following 24 hours of a hypoglycemic reading in the case where the treated hypoglycemic reading was not followed by a retest

Subsequent CBG Reading
In % for each glycaemic range

	Q1: <58			Q2: 58-65			Q3: 66-74			Q4: >74		
# of Participants		n=109			n=99			n=114			n=74	
BG Level	**<3.9**	**3.9-10**	**>10**	**<3.9**	**3.9-10**	**>10**	**<3.9**	**3.9-10**	**>10**	**<3.9**	**3.9-10**	**>10**
**>10**	1.89	14.61	11.73	2.63	17.9	20.44	2.42	18.55	30.25	2.06	17	46.02
**3.9-10**	5.3	43.92	14.47	4.66	28.28	17.88	3.07	21.27	18.37	1.95	11.72	16.85
**<3.9**	0.84	5.15	2.05	0.89	4.62	2.66	0.54	2.89	2.59	0.36	1.8	2.2
**Total**	8.03	63.68	28.25	8.18	50.8	40.98	6.03	42.71	51.21	4.37	30.52	65.07

Initial CBG Reading

Figure S3: The G2G tables for participants with CBG tests between 3 and 6 per day. This shows the results are consistent compared to when the wider participants were included who had lower testing frequency.

(A)

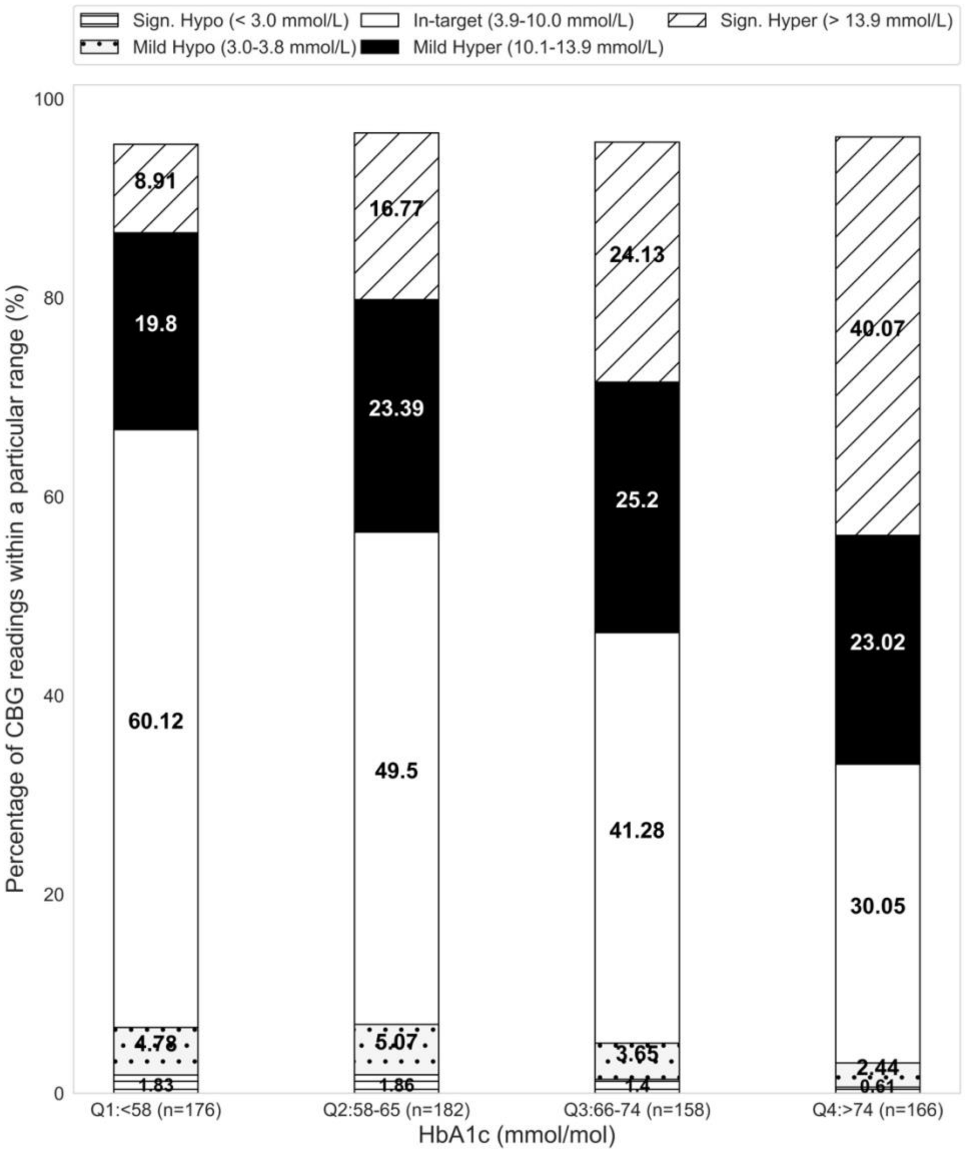

(B)

Subsequent CBG Reading In % for each glycaemic range

	Q1			Q2			Q3			Q4		
BG Levels	**<3.9**	**3.9-10**	**>10**	**<3.9**	**3.9-10**	**>10**	**<3.9**	**3.9-10**	**>10**	**<3.9**	**3.9-10**	**>10**
**>10**	1.48	15.81	11.49	2.61	18.43	19.57	2.37	18.76	29.77	1.68	17.06	44.67
**3.9-10**	4.86	38.25	15.78	3.97	25.53	18.43	2.53	19.13	18.58	1.05	10.46	16.66
**<3.9**	0.40	4.68	1.79	0.44	3.59	2.56	0.15	2.29	2.51	0.0	1.19	1.69
**Total**	6.74	58.74	29.06	7.02	47.55	40.56	5.05	40.18	50.86	2.73	28.71	63.02

Initial CBG Reading

Figure S4: Proportions of 211929 readings in various ranges of CBG for all the participants (Replication of Figure 1 in manuscript but presented in the medians of the percentages instead of mean. (**A**) Percentages of readings across HbA1c quartiles frequency presented in median (**B**) Glucose to glucose (G2G) tables representing the changes between an initial CBG reading in the columns and the next CBG reading in the rows indicated by median of the proportion of readings(%),.
